# What to Observe at the Bed-Side and after Death in Medical Cases

**Published:** 1855-04

**Authors:** 


For sale by MILLER, ORTON & MULLIGAN. H.

---

ART. VII.—*What to Observe at the Bed-side and after Death in Medical Cases.* Published under the Authority of the London Medical Society of Observation. Second American from the second and enlarged London Edition. Philadelphia: Blanchard and Lea. 1855.

Whatever is worth doing at all is worth doing well. Where one examines a patient with a view to scientific inquiry, observations cannot be made too closely, and a system becomes necessary, both that no part may be neglected, and in order that cases of different observers may present some uniformity of arrangement. Errors in diagnosis arise oftener from symptoms overlooked, than from symptoms misinterpreted; and though few men can have the time or patience to observe all their cases in the careful manner here recommended, yet whenever one wishes to light up a doubtful diagnosis, he may here find a remembrancer of symptoms he might otherwise neglect; should he devote attention to a special class of diseases it will aid him in his research; or, finally, in examinations *post-mortem*, whether judicial or medical, it points out all that should be noted, and offers a blank which affords space for every fact.

